# Soft-Tissue Sarcoma following Traumatic Injury: Case Report and Review of the Literature

**DOI:** 10.3389/fonc.2017.00134

**Published:** 2017-06-26

**Authors:** Yael Bar, Ofer Merimsky

**Affiliations:** ^1^Oncology Division, Tel Aviv Sourasky Medical Center, Affiliated with Sackler Faculty of Medicine, Tel Aviv University, Tel Aviv, Israel

**Keywords:** injury, wound, soft-tissue sarcoma, rhabdomyosarcoma, metal fragments, radiation, inflammation

## Abstract

Soft-tissue sarcomas (STSs) are a heterogeneous group of tumors, which accounts for 1–2% of adult cancers worldwide. Despite quite a few reports on traumatic events followed by STS formation, the link between the two events remains a point of controversy. In this paper, we present the case of a young patient who had a rhabdomyosarcoma in the lower extremity, which had developed in the same location where the patient was wounded by a gunshot 9 years earlier. X-ray and CT scans clearly showed metal fragments in the area of sarcoma formation. The patient underwent neoadjuvant chemotherapy treatment, to which the tumor was, unfortunately, unresponsive. Therefore, the patient was referred to below-knee amputation of the injured leg. There are several possible etiological factors for sarcoma development in this patient, including tissue damage and inflammation, as well as the presence of metal fragments in the tissue and the limb’s exposure to radiation during multiple imaging tests. Here, we will discuss the potential influence wielded by the injury itself, as well as its complications and its medical management on the formation of the sarcoma, in light of the current literature.

## Introduction

Soft-tissue sarcomas (STSs) represent a heterogeneous mix of more than 50 mesenchymal types of tumors, which, altogether, encompass approximately 1–2% of adult cancers worldwide (1, 2). The question of whether trauma can cause malignancy in general and sarcoma, in particular, has been preoccupying researchers since the late eighteenth century (3). Nevertheless, despite quite a few reports in the last 100 years on traumatic events followed by STS formation (4–6), the causative relation between the two events remains a point of controversy. Recent works in lab animals show a causative connection between acute tissue injury and sarcoma formation (7, 8).

In this work, we present a case of a young patient with Rhabdomyosarcoma in the lower extremity, who had developed in the same location where the patient was injured by a gunshot 9 years prior. We discuss the potential influence of the injury, its complications, and its medical management on sarcoma formation, in light of the current literature.

## Case Report

A 31-year-old patient was diagnosed with rhabdomyosarcoma of the right calf. Nine years earlier, while in active military service, he was injured by a gunshot wound. Three bullets hit his stomach and led to the destruction of the left kidney, a perforation of the small intestine, and damage to soft tissue in the retroperitoneum. Two more bullets struck the right calf and the right ankle. After he was intubated, an urgent abdominal surgery was performed, which included the removal of the injured kidney. In addition, the patient underwent external fixation of the right fibula and tibia. He was then transferred to the intensive care unit for further treatment.

**Figure 1:**
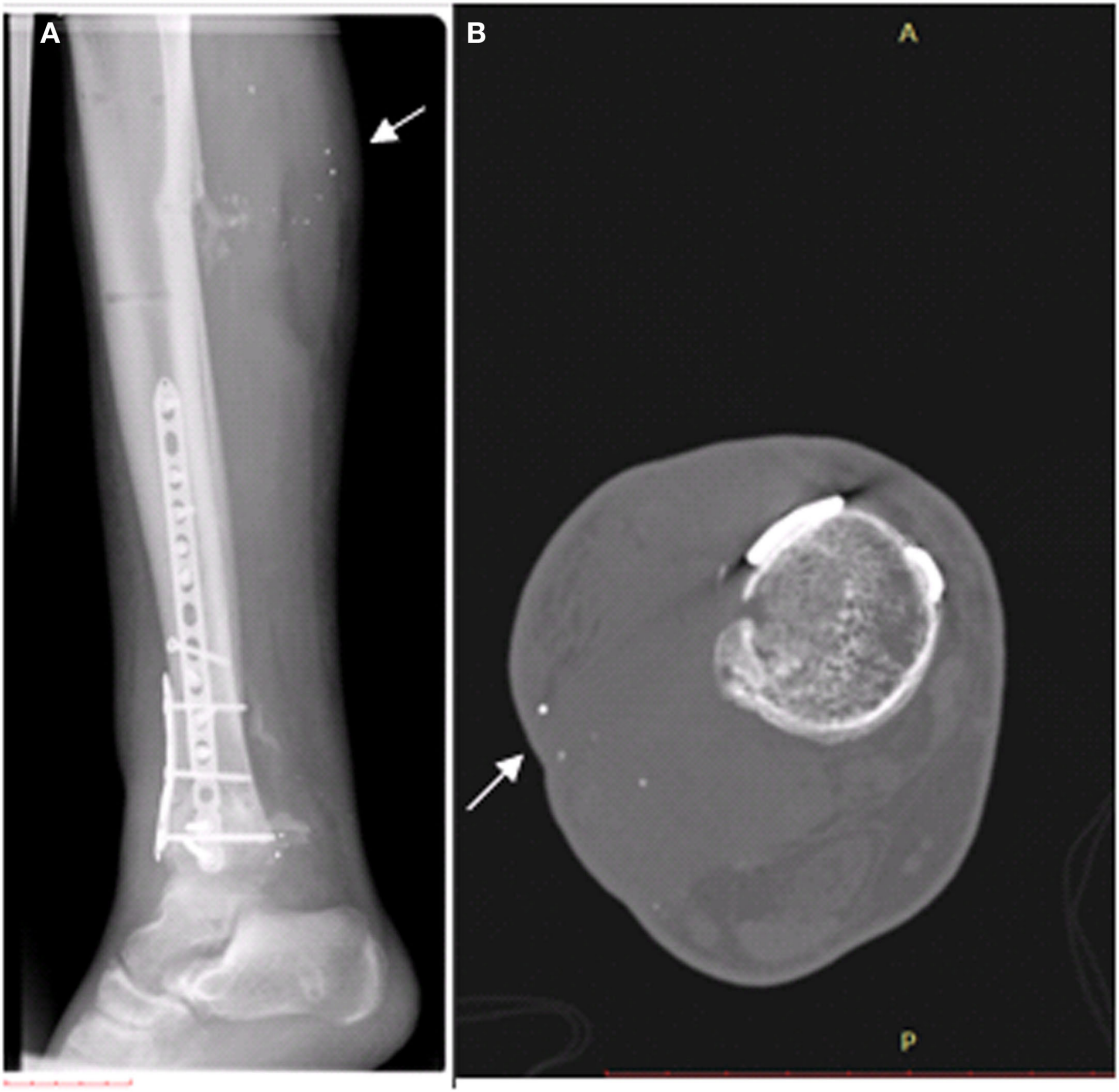
Existence of metal fragments (white arrows), which were not removed during the surgical procedures, in the right calf. **(A)** X-ray preformed 1 year after the injury. **(B)** CT scan preformed 3 years after the injury.

After a month, the patient underwent skin grafting in the area of the gunshot wounds on the right calf and right ankle. Then, the patient was transferred to a rehabilitative department in another hospital. After 2 months, a conversion surgery from external fixation to internal fixation was performed on the right leg. Later, due to an infection of the wound in the right ankle, the patient was treated with antibiotics for 6 weeks and then, due to the continuation of the infection, an additional procedure was performed for the removal of metal fragments from the right ankle. It should be noted that during his hospitalization and follow-up period, the patient underwent dozens of imaging tests for the injured leg, including X-rays, CT scans, and skeletal scans. X-ray and CT scans of the right leg, which were performed for surveillance purposes 1 and 3 years following the injury, clearly indicate the presence of remaining metal fragments, which were not removed during the surgical procedures (Figure 1).

**Figure 2:**
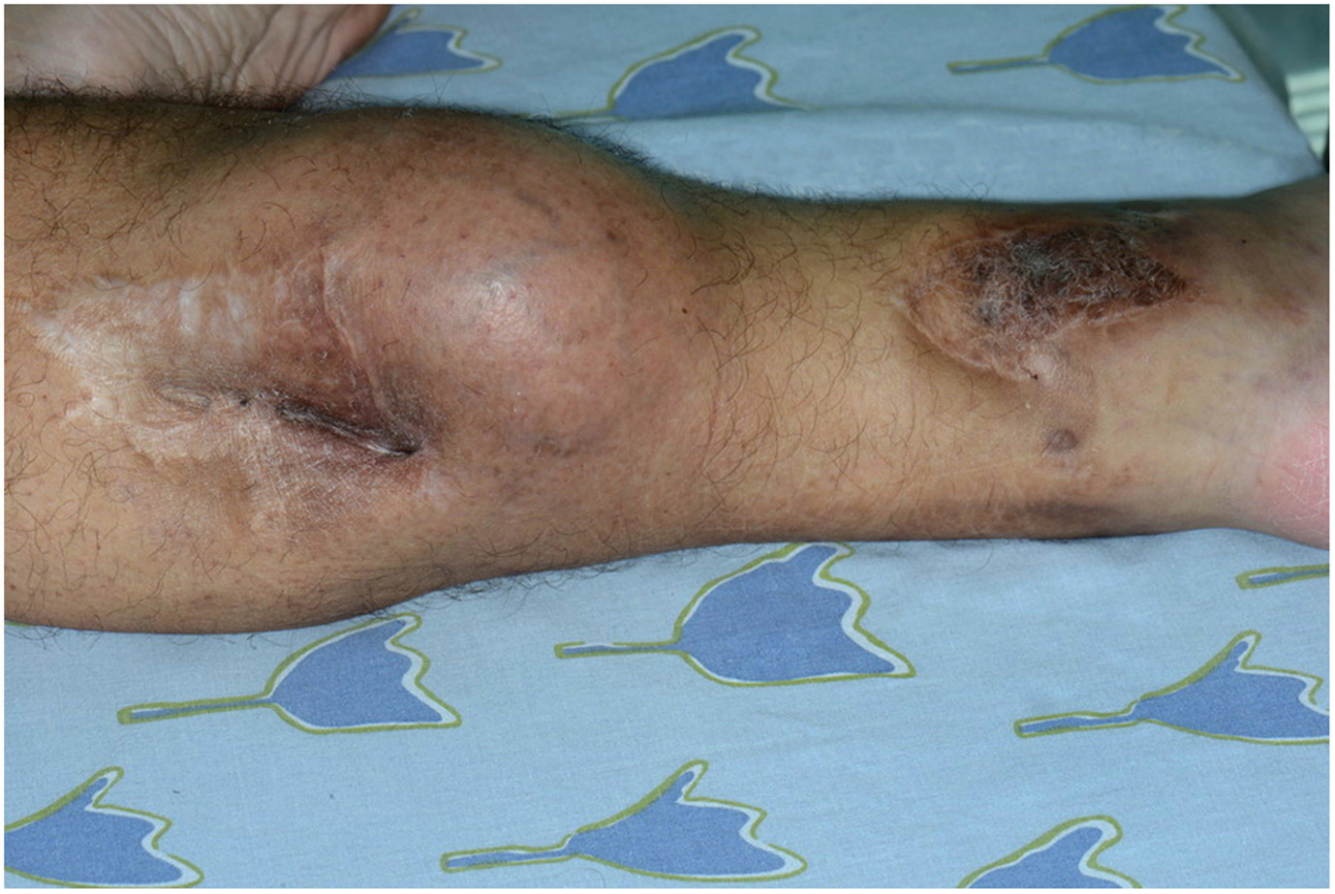
Neoplastic mass next to a shotgun wound scar in the calf (left). Additional shotgun wound scar in the ankle (right).

**Figure 3:**
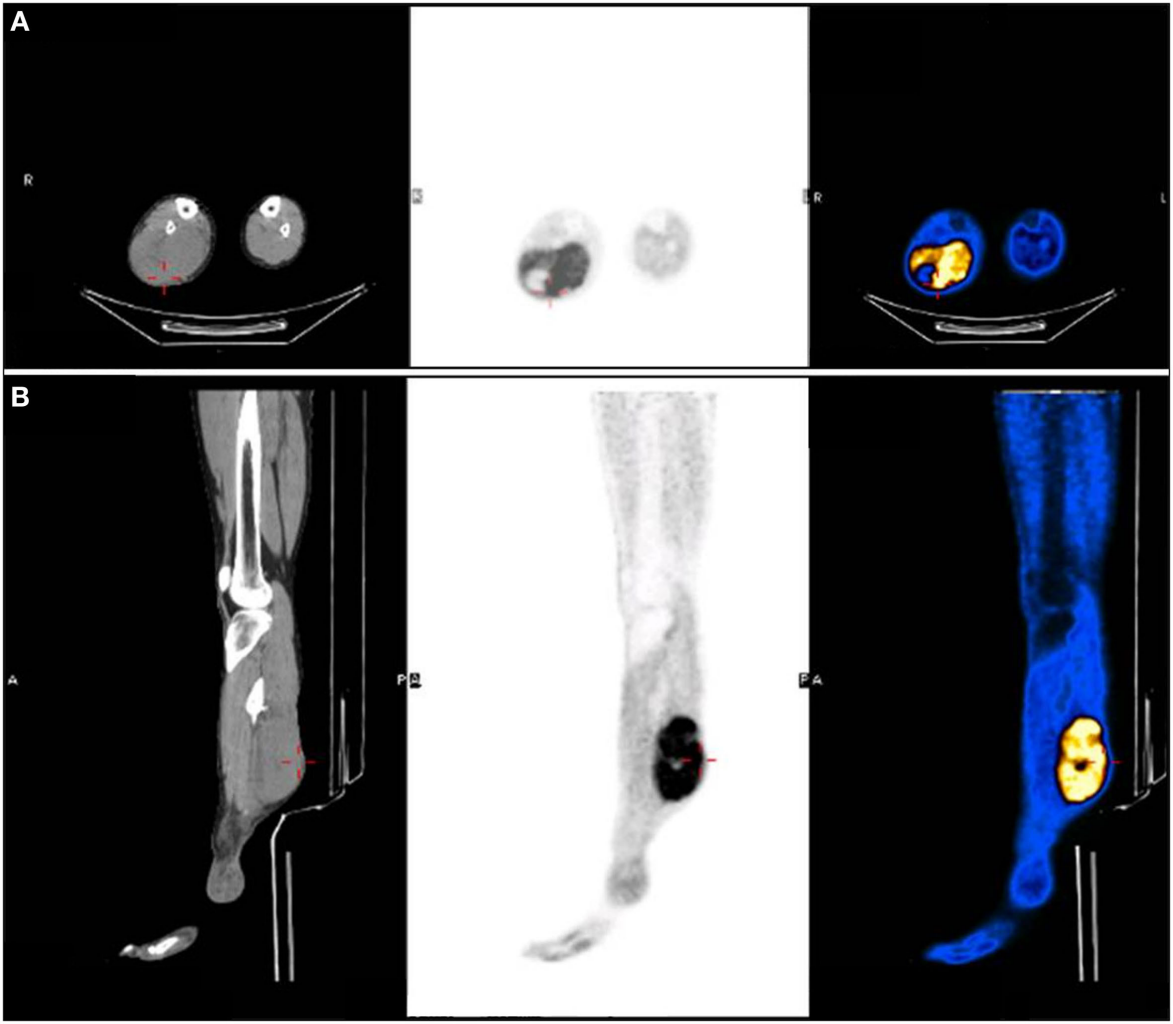
FDG-PET-CT indicated a hypermetabolic mass in the right calf. **(A)** Axial view. **(B)** Sagittal view.

**Figure 4:**
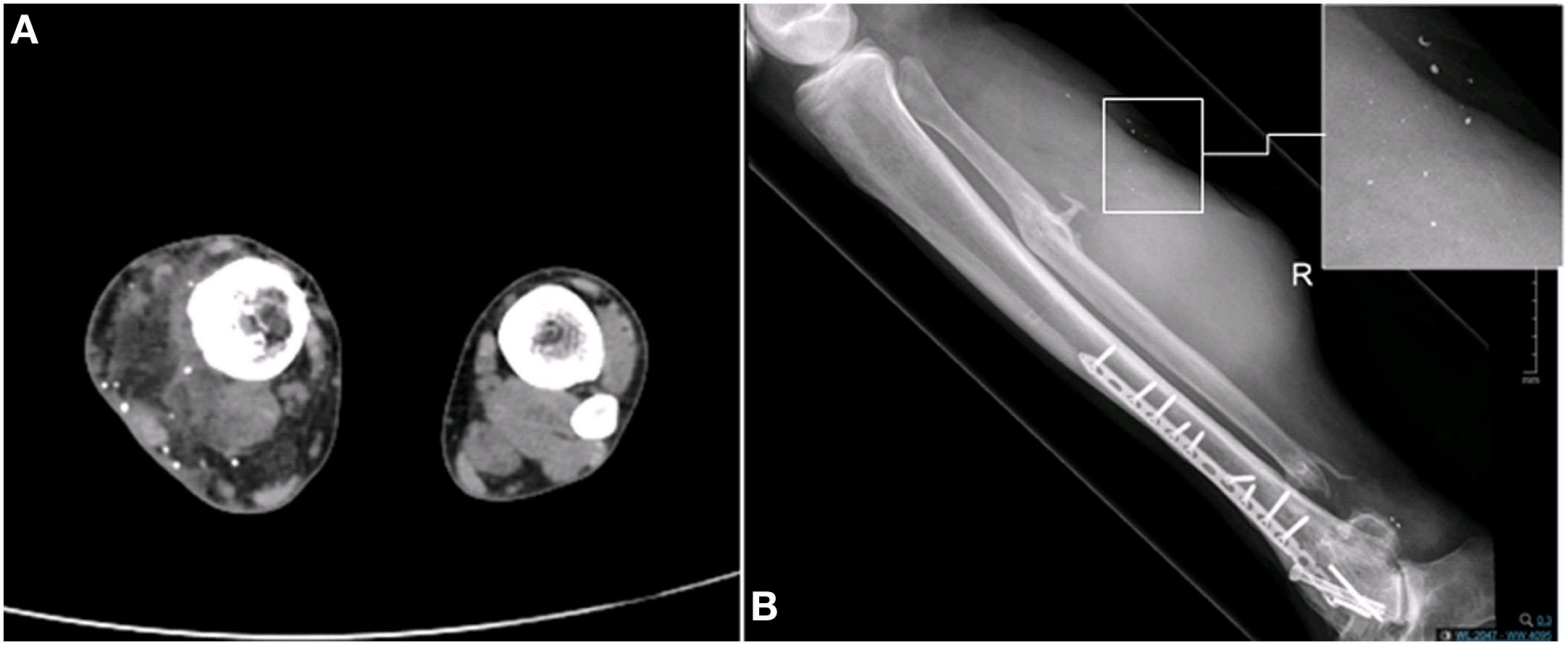
Existence of metal fragments in the area of the sarcoma in the right calf. **(A)** CT scan. **(B)** X-ray.

Nine years following the injury, the patient began to notice a bulge in the area of the surgical scar in the right posterior calf (Figure 2). The patient was referred to a biopsy from the mass and the pathology indicated pleomorphic rhabdomyosarcoma. A FDG-PET-CT indicated a mass in the right calf, another hypermetabolic area near the right ankle; an area of former prolonged infection, and no clear-distance metastatic spread (Figure 3). An X-ray clearly showed metal fragments in the area of the sarcoma in the right calf (Figure 4). Following a multidisciplinary discussion, the patient was referred to neoadjuvant treatment with VAC-IE chemotherapy regiment. A FDG-PET-CT preformed after two chemotherapy cycles indicated that the tumor had no response to the treatment. Hence, the patient was referred to below-knee amputation of the right leg.

## Discussion

The presented case raises the possibility that there is a connection between sarcoma development and tissue injury, 9 years apart in the same location. If this type of connection exists, it is likely derived from combination of several etiological factors: (a) tissue damage and the regenerative and inflammatory processes following it; (b) the presence of metal fragments in the tissue; and (c) radiation to the limb by multiple imaging tests. Below, we review the current knowledge regarding each of these factors.

There are quite a few reports of STS formation following traumatic injury, surgical or other (4–6). Nevertheless, there is a great challenge in confirming a causative relation between the two events. This is partially due to the fact that trauma often draws attention to an already existing mass in the same location. There have been several studies conducted on lab animals that indicated a direct connection between tissue injury and sarcoma formation. Several subsequent works showed that injection of Rous sarcoma virus to chickens resulted in sarcoma development uniquely at the virus injection site in the wing, or in the contralateral wing, after being wounded with a clip. Interestingly, no other tumors were found distant from those sites, despite the systemic presence of the virus in the blood (9–11). Similarly, transgenic mice constitutively overexpressing the V-jun oncogene showed sarcoma formation only at the site of ear tagging and tail clipping (12–14). Transforming growth factor β, an inflammatory growth factor implicated in wound healing, can replace wounding in tumor development in Rous sarcoma virus-infected chicks. Contrastingly, the development of these wound tumors was inhibited by using β-methylprednisolone, an anti-inflammatory drug (11). These results emphasize the role of inflammation and cytokines release in the development of wound tumors. More recently, three published works investigated the connection between direct muscle injury and sarcogenesis in mice that have a genetic predisposition for sarcoma formation. First, Camboni et al. showed that cardiotoxin-induced muscle injury leads to STS formation at the site of injury in p53-null mice (7). In additional work, efforts to generate urothelial tumors in a mouse model harboring kRAS inducible activation, and loss of p53, resulted in STS development at the site of the suture (15). Finally, Van Mater et al. showed that muscle injury by cardiotoxin following specific P53 shutdown and kRAS activation in muscle PAX7+ cells cause accelerated sarcoma formation at the site of injury. Interestingly, no tumor developed when using cardiotoxin injection alone. Hence, the authors propose an initiator/promoter model, whereby injury acts as the promoter to drive sarcoma formation after the initiating genetic insult (8).

The link between tissue injury and sarcoma formation could be derived from the known link between inflammation and tumorigenesis (16–19). It is known that wound healing response to injury, triggers functional and phenotypic changes in fibroblasts, lymphocytes, epithelial, and endothelial cells. This is a dynamic process that consists of an inflammatory phase followed by epithelial cell proliferation and tissue remodeling (20, 21). Failure to exit the inflammatory stage results in improper tissue remodeling and chronic inflammation. Chronic inflammation and the secretion of inflammatory mediators by inflammatory cells (cytokines, chemokines, and growth factors) has been linked to tumorigenesis, tumor progression, and metastasis in many different cancers (16, 17, 19) including sarcomas (22).

During the treatment to the injured leg, the presented patient underwent multiple imaging examinations, including X-ray, CT, and bone scans. There is a well-established causative connection between radiation exposure and STS. STSs are one of the most common types of radiation-associated cancers in the general population (23–26). The median interval between radiotherapy and development of a radiation-associated sarcoma is about 10 years (27). This interval varied significantly by histologic type. Undifferentiated pleomorphic sarcomas constitute the most common type of radiation-induced sarcoma. Though uncommon, radiation-induced sarcomas usually have a poor prognosis (27). The molecular mechanisms of radiation-associated sarcomas are poorly understood (28).

The injured leg CT and X-ray images clearly show metal fragments in the area of the developing mass (Figure 4). Several works showed a connection between the presence of metal fragments in the tissue and sarcogenesis. Triggered by the usage of depleted uranium (DU) in the Gulf War, a work by Hahn et al. checked the carcinogenicity of DU metal in the muscle tissue of rats. In this work, DU particles cause localized proliferative reactions and formation of STS. The mechanism by which DU particles induced sarcomas is uncertain; however, the authors suggested that the corrosion of the particles in the tissue and the inflammatory response to the particles might lead to sarcoma formation (29). The composition of the bullets has changed over the years and now includes other metals such as tungsten, nickel, cobalt, and iron instead of uranium. Implantation of pellets containing tungsten, nickel and cobalt, into the quadriceps muscle of mice led to the development of a rhabdomyosarcoma around the pellet (30). Similarly, when implanted into the leg muscle of F344 rats, tungsten/nickel/cobalt pellets were found to induce highly aggressive metastatic rhabdomyosarcomas within 6–12 months (31, 32). Progressive corrosion of the pellets was clearly observed by electron microscopy. A microarray gene analysis of the tumors compared to normal muscle, revealed significant upregulation of cell cycle-associated genes as well as significant downregulation of muscle development and differentiation associated genes (32). Interestingly, the histological features of the sarcomas developed in rats following pellets implantation (31), high-grade pleomorphic rhabdomyosarcoma, was similar to the histological features of our patient’s sarcoma.

## Conclusion

The case presented above suggests the existence of a link between gunshot injury and the development of STS at the same location 9 years later. Although there are previous reports on sarcoma formation following trauma, this work places an emphasis on the different aspects of the traumatic event that can lead to sarcoma formation. This includes the injury itself, as well as the regenerative and inflammatory processes it entails, alongside its related complications and, of equal importance—its medical management and treatment. More work is needed to establish the connection between traumatic injury and STS formation and to determine the biological processes underline this phenomenon.

## Ethics Statement

We confirm that the patient gave written informed consent for the publication of the report and for the publication of the picture presented in Figure 2.

## Author Contributions

OM: treated the patient. YB: wrote the paper and reviewed the literature.

## Conflict of Interest Statement

The authors declare that the research was conducted in the absence of any commercial or financial relationships that could be construed as a potential conflict of interest.
